# Differential Control of Notch1 Gene Transcription by Klf4 and Sp3 Transcription Factors in Normal versus Cancer-Derived Keratinocytes

**DOI:** 10.1371/journal.pone.0010369

**Published:** 2010-04-28

**Authors:** Chiara Lambertini, Serafino Pantano, G. Paolo Dotto

**Affiliations:** 1 Department of Biochemistry, University of Lausanne, Epalinges, Switzerland; 2 Cutaneous Biology Research Center, Massachusetts General Hospital, Charlestown, Massachusetts, United States of America; Brunel University, United Kingdom

## Abstract

In specific cell types like keratinocytes, Notch signaling plays an important pro-differentiation and tumor suppressing function, with down-modulation of the Notch1 gene being associated with cancer development. Besides being controlled by p53, little else is known on regulation of Notch1 gene expression in this context. We report here that transcription of this gene is driven by a TATA-less “sharp peak” promoter and that the minimal functional region of this promoter, which extends from the −342 bp position to the initiation codon, is differentially active in normal versus cancer cells. This GC rich region lacks p53 binding sites, but binds Klf4 and Sp3. This finding is likely to be of biological significance, as Klf4 and, to a lesser extent, Sp3 are up-regulated in a number of cancer cells where Notch1 expression is down-modulated, and Klf4 over-expression in normal cells is sufficient to down-modulate Notch1 gene transcription. The combined knock-down of Klf4 and Sp3 was necessary for the reverse effect of increasing Notch1 transcription, consistent with the two factors exerting an overlapping repressor function through their binding to the Notch1 promoter.

## Introduction

Notch signaling plays a key role in control of cell fate determination, growth and differentiation [1], [2]. It is also a determinant of carcinogenesis, with either a positive or negative role depending on cell type and specific context [3], [4]. The best characterized “canonical” pathway of Notch activation involves proteolytic cleavage and translocation of the cytoplasmic domain of the receptor to the nucleus, where it associates with the DNA binding protein CSL converting it from a repressor into an activator of transcription [1], [2]. Most attention has been given to control of the Notch pathway at the post-transcriptional level, including processing and maturation of Notch receptors, activation by ligands at the cell surface, and protein modification and degradation [1], [2]. However, a main form of regulation can also be at the level of gene transcription. Expression of the four Notch receptor genes and their ligands is regulated in specific cellular contexts and can be altered in cancer development [3], [5]. Furthermore, activation of one receptor is susceptible of affecting its own expression as well as of other family members, and can also impinge, in a positive or negative manner, on ligand expression [1], [2].

An example of this complex mode of regulation of Notch expression has been provided by studies in keratinocytes, where this pathway plays a key role in promoting differentiation and suppressing tumorigenesis [3]. In the basal layer of the epidermis, reciprocal negative regulation between ligands of the Delta family and Notch receptors has been proposed to control the balance between putative keratinocyte stem cell populations and cells committed to differentiation [6]. On the other hand, positive feedback regulation between Notch receptors and ligands of the Jagged family has been implicated as a possible mechanism for synchronization of the keratinocyte transition from the basal proliferating to suprabasal differentiating layers of the epidermis [7]. While both Notch1 and Notch2 receptors are expressed in the interfollicular epidermis, their regulation and function are only partially overlapping. In particular, while Notch2 levels are uniformly elevated in the differentiating layers of the epidermis, expression of Notch1 is shut off in the outermost layers [7]. This may be of functional importance, since elevated Notch1 activity, while required for commitment and entry into differentiation, can then suppress the latest steps of this process [7], [8]. Similarly, in keratinocyte-derived tumors, like cutaneous squamous cell carcinomas (SCCs), basal cell carcinomas (BCCs) [9], [10] and late stage cervical carcinomas [11], Notch1 expression is decreased to a substantially greater extent than Notch2. This is of likely functional significance as, even in the presence of Notch2, deletion of the Notch1 gene is by itself sufficient to promote keratinocyte tumor development [9], [11], [12].

Surprisingly little is know on control of Notch1 gene expression. In keratinocytes, endogenous p53 binds to the Notch1 promoter, increasing its transcription, and compromised p53 function can explain, at least in part, the tumor-associated down-modulation of Notch1 expression [9], [13], [14]. Similarly, decreased p53 levels via viral E6-dependent degradation can explain the already mentioned down-regulation of Notch1 expression in HPV-positive cervical carcinoma cells [14]. Control of Notch1 expression by p53 is also of relevance for the keratinocyte response to UV light, a major etiological agent of skin aging and cancer [13], [14]. Besides keratinocytes, and their malignant counterparts, Notch1 expression is under positive p53 control in lung and prostate cancer cells, other cell types in which increased Notch signaling causes growth suppression [15], as well as in B-cell chronic lymphocytic leukemia cells, where Notch signaling enhances cell survival [16]. In many of these cases, p53 was found to be specifically involved in control of the Notch1 gene with little or no effects on other family members [9], [14], [15]. Interestingly, no such regulation was found in colon carcinoma cells, in spite of binding of p53 to the Notch1 promoter even in these cells, pointing to the likely interplay between p53 and other as yet unidentified determinants of Notch1 gene transcription [9].

The Sp/KLF family of transcription factors consists of proteins with three highly conserved DNA-binding zinc finger domains, which recognize GC/CACCC boxes present in many GC-rich promoters [17]. These factors play an important role in transcription of housekeeping genes as well as genes with more restricted cell type specific functions [18]. Among Klf family members, Klf4 has attracted recent attention because of its capability, in concert with other factors, to reprogram cell fate determination [19], as well as promoting or suppressing cancer development in a context dependent manner [20]. We report here that in keratinocytes Klf4 binds to the Notch1 promoter and, together with Sp3, functions as a negative regulator of Notch1 gene transcription, affecting recruitment of the PolII preinitiation complex through a separate mechanism from p53. Inhibition of Notch1 expression by Klf4 is consistent with the essential role of Klf4 in the late stages of keratinocyte differentiation [21], for which expression of the Notch1 gene needs to be turned off [7]. However, the same regulatory mechanism can also contribute to down-modulation of Notch1 expression in keratinocyte-derived tumors, where Klf4 can be aberrantly over-expressed.

## Results

### 1. Transcription of the Notch1 gene from a ‘sharp peak’ TATA less promoter

**Figure 1:**
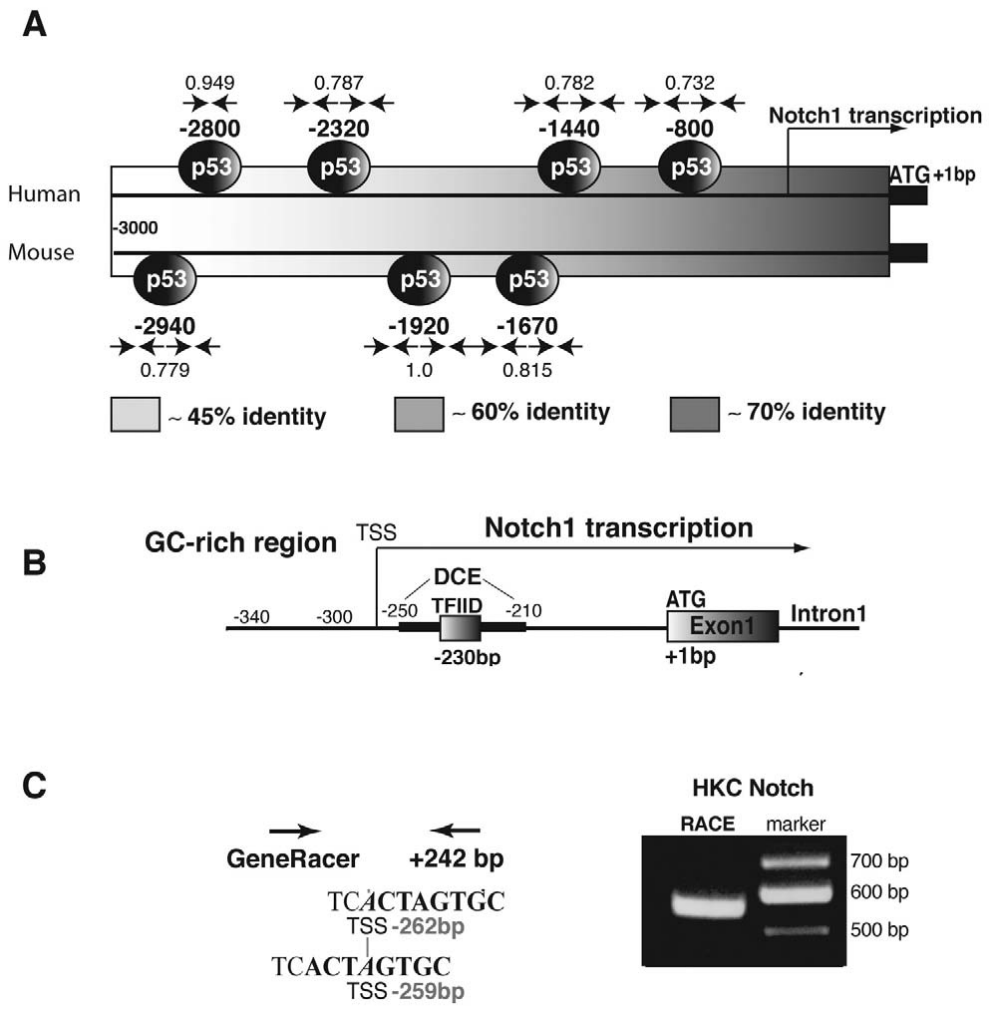
Characterization of the upstream region of the Notch1 gene. (A) Nucleotide sequence comparison of the 3.0 kb upstream region of the human and mouse Notch1 genes, with increasing homology towards the coding region illustrated by a grey intensity gradient. The position of putative p53-binding sites, as predicted by a bioinformatics program (MatInspector, http://www.genomatix.de) is indicated, relative to the initiation codon. “Canonical” p53-binding sites are composed of two half-sites with the nucleotide sequence RRRCA/T-T/AGYYY (with R  =  purine; and Y  =  pyrimidine), separated by a spacer of 0–21 nucleotides. Quarter-sites (RRRCW and WGYYY) can be present in a head to head, or head to tail orientation, as indicated by arrows (→ or ←). Numbers above and below refer to the matching score between the nucleotide sequence of each site and the predicted matrix, with a perfect match = 1.00, and a “good” match >0.80. (B) Core promoter elements of the human Notch1 gene with an indication of the GC-rich proximal region, putative TFIID binding site and distal core elements (DCE) [53] (as identified by the TESS program) and Notch1 transcription start sites (TSS). (C) Experimental determination of the Notch1 TSS by 5′ RACE. Human primary keratinocytes were used as source of total RNA for 5′ RACE amplification using GeneRacer and a specific reverse primer located at +242 bp from the initiation codon. Cloning and sequencing of the amplification reaction product (as shown after gel electrophoresis) pointed to a major TSS at position −262 and a second at position −259. The sequence of overlapping initiator elements (Py Py A(+1) N T(+3) Py Py), in our case TCA(+1)CT(+3)AG for the major TSS, and CTA(+1)GT(+3)GC for the second TSS, is indicated in bold, while the first nucleotides of the two TSS are in Italics.

Besides the involvement of p53 [9], [13], [14], [22], little is known on control of Notch1 gene transcription. The human Notch1 gene is located on chromosome 9 and structured in 34 exons, which code for a large protein of about 270 kDa. For further insights into transcriptional control of this gene, we compared its upstream non-coding sequence in the human versus mouse genomes. Several predicted p53 binding sites are present in the 3.0 kb upstream sequence of the mouse and human Notch1 promoters although at different positions relative to the initiation codon (Fig. 1A). A gradient of increasing sequence homology was found towards the human and mouse initiation codons, with >70% of sequence identity in the GC-rich domain from position −500 bp to the ATG. Nucleotide analysis further pointed to a putative TFIID-binding site around position −230 bp surrounded by distal core elements, while no TATA boxes could be identified (using the TESS, Consite or Genomatix programs; www.cbil.upenn.edu/cgi-bin/tess/tess; asp.ii.uib.no:8090/cgi-bin/CONSITE/consite; www.genomatix.de) (Fig. 1B).

To establish experimentally the transcription start site (TSS) of the Notch1 transcript in primary human keratinocytes (HKC), we cloned and sequenced the products of 5′-RACE (Rapid amplification of 5′ complementary DNA ends) reaction from these cells (Fig. 1C). The results pointed to a main TSS of the human Notch1 gene at position −262 bp from the ATG, with a second minor TSS at −259 bp. Thus, transcription of the Notch1 gene is driven by a CpG island promoter, which, unlike most promoters of this class [23], has the characteristics of a ‘sharp peak’ promoter, with precise TSSs probably determined by an overlapping initiator (Inr) element [24].

### 2. Mapping of the functional Notch1 promoter region in normal versus cancer cells

**Figure 2:**
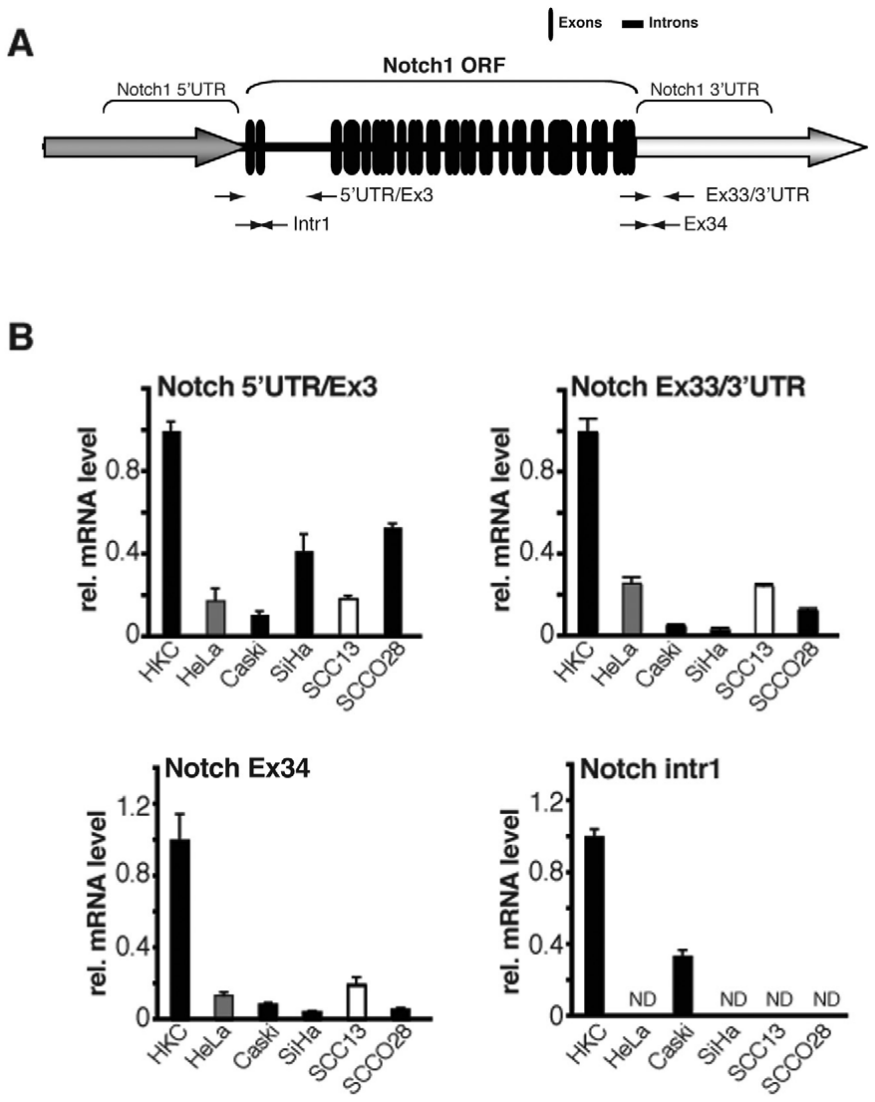
Transcription of various regions of the Notch1 gene in primary human keratinocytes versus keratinocyte-derived cancer cell lines. (A) Organization of the Notch1 gene, with an indication of the open reading frame (ORF) with coding exons and intervening introns (black thick boxes and lines, respectively) and 5′ and 3′ untranslated regions (UTR). The position of the different sets of primers utilized for real time RT-PCR analysis is also indicated (thin arrows). (B) Total RNA from primary human keratinocytes (HKC), cervical carcinoma cells (HeLa, Caski and SiHa), and skin (SCC13) and oral (SCCO28) squamous carcinoma cells was analyzed by real-time RT-PCR with primers corresponding to different region of the Notch1 gene as indicated in (A). For this and all other figures, values were normalized to 36B4 and/or 18S RNA levels, and expressed as relative to those in primary keratinocytes.

mRNA expression can be controlled at multiple levels, from the first steps of transcription (initiation/elongation) to RNA processing and stability [25]. Real time RT-PCR analysis with primers specific for the 5′ and 3′ regions of the Notch1 transcript and first intron showed that the higher expression of Notch1 mRNA previously reported in HKC versus keratinocyte-derived cancer cells [9], [11] was maintained irrespectively of the specific regions of the Notch1 transcript that were analyzed (Fig. 2).

**Figure 3:**
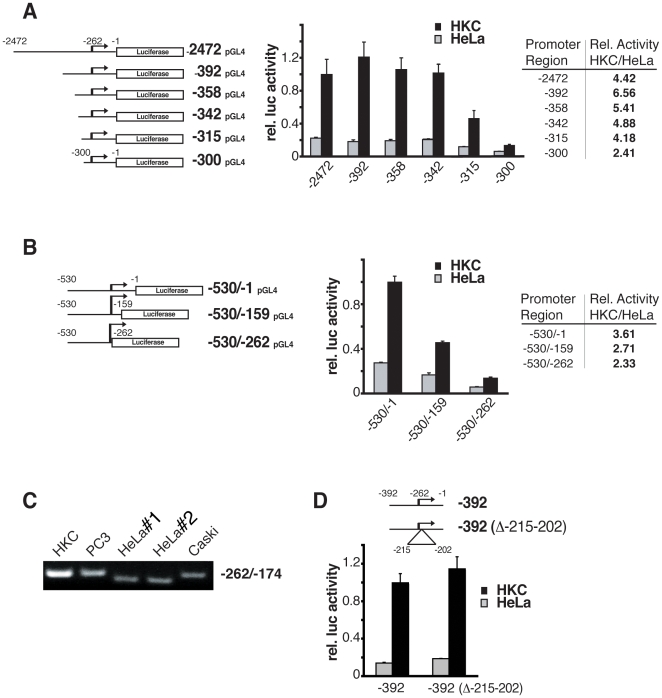
Mapping of the minimal functional region of the Notch1 promoter in primary human keratinocytes versus HeLa cells. (A) Different fragments of the human Notch1 promoter with decreasing 5′ ends were cloned into a luciferase reporter plasmid, followed by transient transfection into primary human keratinocytes and HeLa cells (black and grey bars, respectively) together with a Renilla minimal reporter for normalization. Promoter activity was measured 48 hours after transfection. Relative promoter activity of the various reporters in HKC versus HeLa was also calculated (right Table). (B) Fragments of the human Notch1 promoter with partial or total deletion of the sequence from the TSS to the initiation codon (lacking nucleotides −159 to −1, and −262 to −1, respectively) were cloned into a luciferase reporter plasmid, followed by transient transfection/promoter activity assays in primary human keratinocytes and HeLa cells as in the previous panel. Relative promoter activity in HKC versus HeLa was also calculated. (C) Total RNA from primary keratinocytes and various cancer cells lines, including PC3, Caski, and HeLa from two different sources (HeLa#1 and 2), was utilized for RT-PCR amplification of the 5′ UTR region of the Notch 1 gene (primer position −262/−174). Note the faster migrating band obtained with the HeLa samples. Cloning and sequencing of the overlapping genomic region (from position −392 bp to −1 of the Notch1 promoter) showed a deletion from position −215 to −202 in HeLa cells. (D) The same Notch1 genomic region plus/minus the above deletion was cloned into a luciferase reporter plasmid, followed by promoter activity assays in HKC versus HeLa cells, measured as in (A) and (B).

These results suggested that transcription of the Notch1 gene is differentially controlled in normal versus cancer cells already at the initial steps of transcription. To further test this possibility, we determined the minimal functional region of the Notch1 promoter and assessed whether such region is differentially active in normal versus cancer cells. For this purpose, the 2.4 kb region of the Notch1 promoter upstream of the initiation codon was cloned into a luciferase reporter, followed by promoter activity assays in HKCs versus HeLa cells. Testing a series of fragments with progressively reduced length from the 5′ end showed that a 342 bp region from the initiation codon retains full promoter activity in HKCs, while further shortening of the Notch1 promoter to −315 bp and −300 bp regions resulted in progressively reduced activity (Fig. 3A). Notch1 promoter regions with full activity in HKCs were significantly less active in HeLa cells, reflecting the differences in endogenous Notch1 gene transcription, while the difference in promoter activity became less with shorter Notch1 promoter fragments (Fig. 3A).

Nucleotide sequences downstream of transcription start sites (TSS) play also an important role in control of promoter activity, being required for the formation of the transcription pre-initiation and initiation complexes [26]. Consistent with the importance of this region, promoter activity was substantially decreased when the sequence of the Notch1 promoter downstream of the TSS to the initiation codon (from −262 to −1) was partially or totally deleted (with promoter constructs lacking nucleotides −159 to −1, and −262 to −1, respectively) (Fig. 3B). Even in this case, intrinsically decreased promoter activity was accompanied by a lesser difference between normal and cancer cells (Fig. 3B).

PCR amplification of genomic DNA, aimed at assessing its methylation state as shown further below, revealed the existence of a small deletion in the Notch1 promoter region of two different strains of HeLa cells, which was not present in other cancer cells like PC3 and Caski (Fig. 3C). By cloning and sequencing this region, we mapped the deletion to the −215 to −202 bp position, just downstream of the TSS. Functional promoter activity assays of the cloned promoter region promoter in HKC versus HeLa cells showed that this 10 nucleotides deletion did not affect promoter activity nor its differential regulation in normal versus cancer cells (Fig. 3D).

### 3. Negative control of Notch1 gene transcription by Klf4 and Sp3

**Figure 4:**
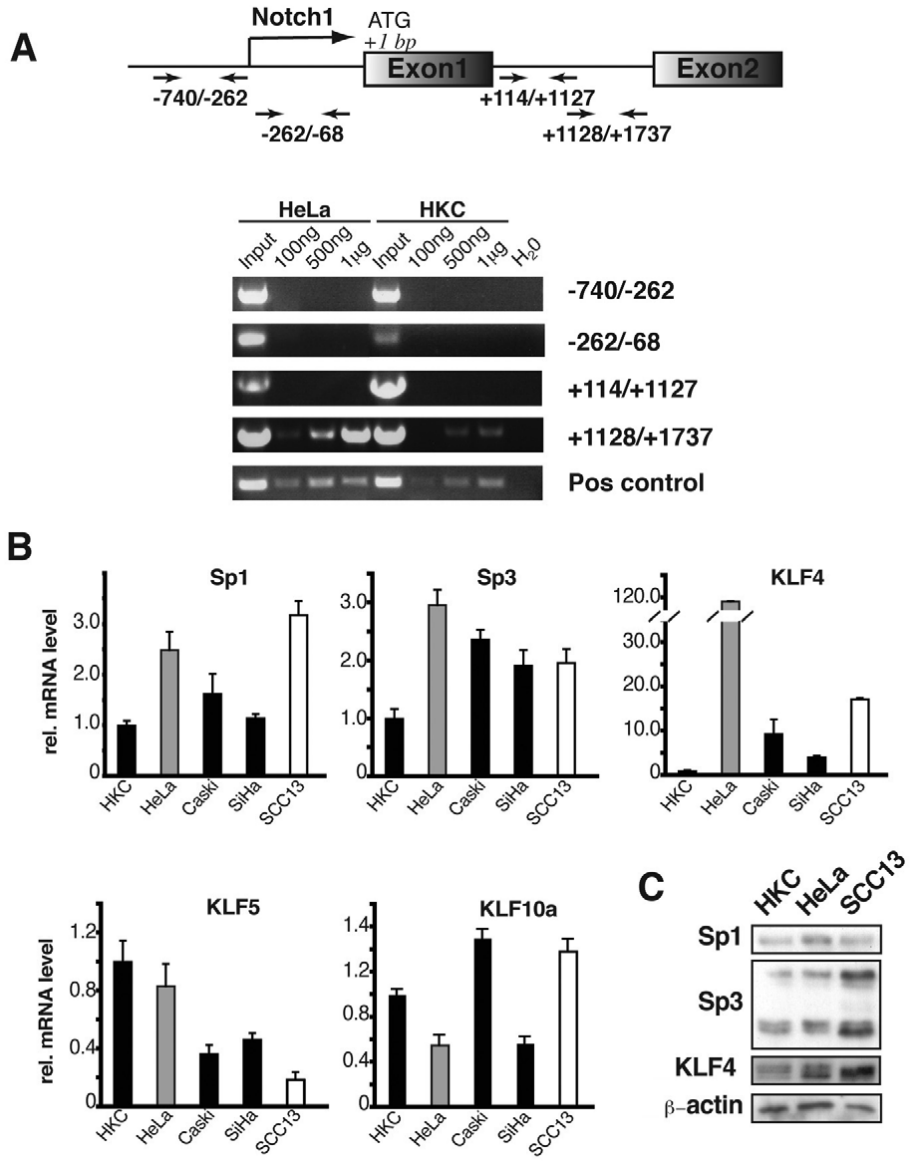
Methylation state of the Notch1 regulatory region and expression of various Sp/KLF family members in primary keratinocytes versus keratinocyte-derived cancer cell lines. (A) Nuclear extracts from primary human keratinocytes (HKC) and HeLa cells were immunoprecipitated with antibodies against methylated DNA, followed by PCR analysis of the precipitated DNA with primers specific for the indicated regions of the Notch1 promoter and first intronic region. PCR with primers specific for a known methylated gene was used as positive control. (B) Total RNA samples from primary human keratinocytes (HKC) and the indicated cancer cell lines were analyzed by real time RT-PCR with primers specific for Sp1, Sp3, Klf4, KLF5 and KLF10a. (C) Primary keratinocytes, HeLa and SCC13 cells were analyzed by immunoblotting with antibodies against Sp1, Sp3 and Klf4, with β-actin as equal loading control.

The differential transcription activity of the GC-rich proximal region of the Notch1 promoter may be linked to a different methylation state in HKCs versus HeLa cells. Pull down assays with antibodies against methylated DNA followed by PCR amplification pointed to a substantial level of methylation of the first intronic region (between nucleotides +1168/+1798) in Hela cells but not in HKCs, which could contribute to different transcription of the Notch1 gene (Fig. 4A). However, no detectable methylation was found in the Notch1 promoter region that may account for its different levels of activity in the two cells. Another possibility is that the differential Notch1 promoter activity is linked to transcription factors that bind to this region and control its activity. Transcription factors with the highest probability to bind to GC rich domains like the one present in the Notch1 promoter proximal region are zinc-finger proteins of the Sp- and KLF families [27]. One or more of these factors may be differentially expressed in normal versus cancer cells. In fact, real time RT-PCR of HKCs versus a panel of cervical carcinoma and keratinocyte-derived SCC cells showed that, of the several Sp/KLF family members that were examined, Klf4 was strongly and consistently up-regulated in cancer cells (Fig. 4B). Sp1 and Sp3 were also up-regulated in these cells, although to a lesser extent than Klf4, while expression of other family members, like KLF5, or KLF10a was only slightly affected and/or down-modulated (Fig. 4B). Lesser differences were observed at the protein level, probably as the result of post-transcriptional and/or protein destabilization events (Fig. 4C).

**Figure 5:**
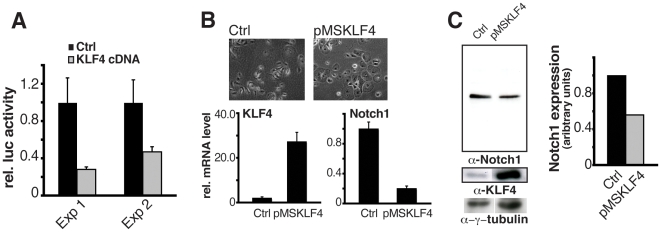
Negative control of Notch1 gene expression by Klf4. (A) Primary keratinocytes were co-transfected with a reporter containing the minimal functional Notch1 promoter (from -392pGL4) together with an expression vector for human Klf4 or empty vector control. Renilla minimal reporter was used for internal normalization, and the promoter activity was measured 48 hours after transfection. Shown are the results of two different experiments. (B) Primary keratinocytes were infected with a retroviral vector expressing KlfF4 (pMSKlf4) or an empty vector control and harvested after 48 hours, followed by determination of Klf4 and Notch1 mRNA expression by real-time PCR. Efficiency of infection with the pMSKlf4 virus was also assessed by the widespread morphological changes with flattening of cells (upper panels). (C) Primary keratinocytes were infected with a KlfF4 expressing retrovirus versus an empty vector control as in the previous panel, followed by immunoblot analysis with antibodies against Notch1, Klf4 and γ-tubulin as equal loading control. Right panel: data were quantified by densitometric scanning of the autoradiograph and expressed as arbitrary units after normalization for γ-tubulin expression. Similar results were obtained in a second independent experiment.

To assess the functional consequences of increased Klf4 expression on Notch1 transcription, two complementary approaches were undertaken. In the first, HKCs were transiently transfected with a Notch1 reporter; promoter activity was suppressed by concomitant transfection with a Klf4 expression vector (Fig. 5A). As a second approach, HKC were infected with a Klf4 expressing retrovirus. Real time RT-PCR as well as immunoblot analysis showed that endogenous Notch1 expression was significantly reduced in these cells as a consequence of Klf4 over-expression (Fig. 5B, C).

**Figure 6:**
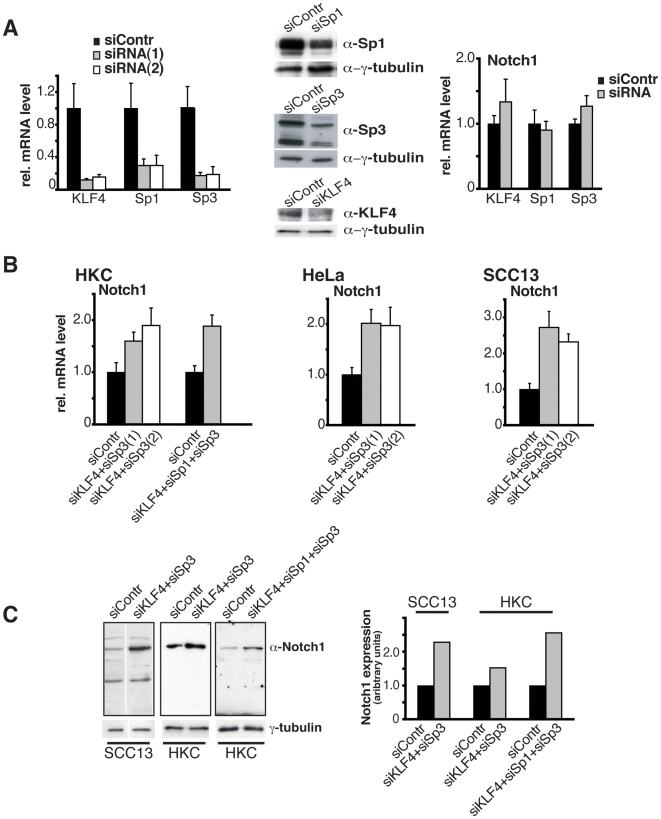
Up-regulation of Notch1 gene expression by Klf4 and Sp3 knock down. (A) Primary keratinocytes were transfected with two sets of siRNAs for Klf4, Sp1 or Sp3 in parallel with scrambled siRNA controls for 48 hours, followed by expression analysis of the targeted genes by real time RT-PCR and immunoblotting (left and middle panels, respectively) The same RNA samples were also analyzed for levels of Notch1 expression (right panel). (B) Primary keratinocytes, were transfected as in the previous panel with two different sets of siRNAs for Klf4 and Sp3 (siRNA-1, siRNA-2) (left columns) or with siRNAs for Klf4, Sp1 and Sp3 (right columns), followed by real time RT-PCR analysis of Notch1 expression. Shown is the calculated average of four different experiments using 36β4 and 18S RNA for internal normalization. (C) HeLa and SCC13 cells were transfected with two different sets of siRNAs for Klf4 and Sp3 (siRNA-1, siRNA-2) followed by determination of Notch1 expression by real-time RT-PCR as in the previous panel. Primary keratinocytes and SCC13 cells were transfected with siRNAs against the indicated genes followed by immunoblot analysis of Notch1 protein expression with γ-tubulin as equal loading control. Right panel: data were quantified by densitometric scanning of the autoradiograph and expressed as arbitrary units after normalization for γ-tubulin expression.

Conversely, to test whether Klf4 suppression leads to Notch1 up-regulation, HKC as well as HeLa cells were transfected with siRNAs for Klf4. Efficient down-modulation of this gene had no effects on Notch1 gene transcription, and similar lack of effects was observed after knock-down of Sp3 and Sp1 (Fig. 6A and data not shown). However, the combined siRNA-mediated knockdown of Klf4 and Sp3 resulted in consistent up-regulation of Notch1 expression in both HKCs and cancer cells (HeLa and SCC13 cells), with the concomitant knock-down of Sp1 having no additional effects (Fig. 6B, C and data not shown).

**Figure 7:**
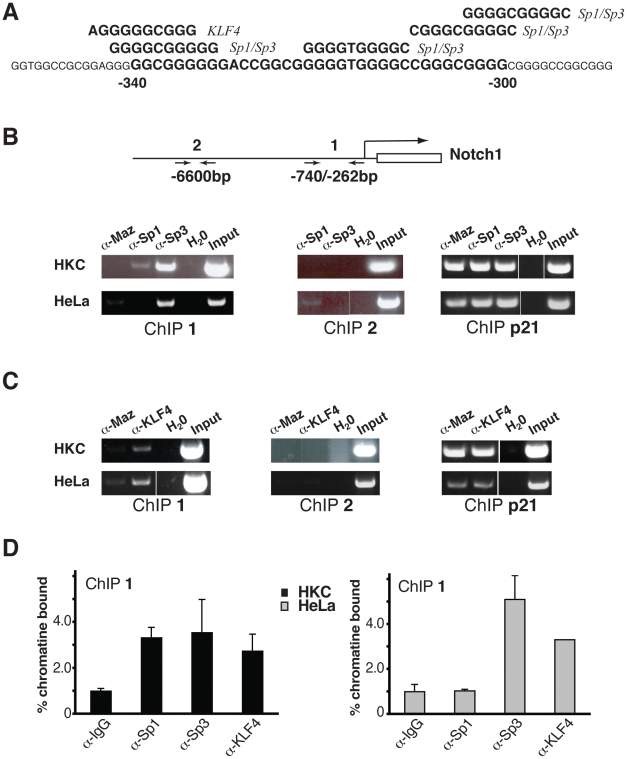
Binding of endogenous Klf4 and Sp1/Sp3 proteins to the Notch1 promoter. (A) Predicted Klf4- and Sp1/Sp3 binding sites in the −340/−300 bp Notch1 promoter region. Shown is the nucleotide sequence of this region with, on top, the predicted binding sites for Klf4- and Sp1/Sp3. (B and C) Primary keratinocytes (HKC) and HeLa cells were processed for chromatin immunoprecipitation (ChIP) analysis with antibodies against Sp1, Sp3 (B), Klf4 (C) or Maz, as indicated, followed by amplification of two Notch1 promoter regions located between bp −740/−262 (Chip 1) and around −6600 bp (Chip 2), which contain and lack, respectively, putative Sp1/Sp3 and Klf4 binding sites. Amplification of the proximal promoter region of the p21^WAF1/Cip1^ gene (Chip p21) was used as positive control. Un-precipitated chromatin preparations were used for parallel amplification reactions as ‘input’ DNA controls. (D) Chip assays with anti-Sp3 and Klf4 antibodies and non immune IgGs as in the previous panels were followed by real time PCR amplification of region1 of the Notch1 promoter. The amount of precipitated DNA was calculated relative to the total input chromatin and expressed as a percentage of the total according to the following formula [54]: percentage total = 2ΔCt×5, where ΔCt  =  Ct (input) − Ct (immunoprecipitation), and Ct is the cycle threshold.

The required combined knockdown of Klf4 and Sp3 for up-regulation of Notch1 expression may be explained by the concomitant binding of these factors to the Notch1 promoter. In fact, chromatin immunoprecipitation (ChIP) assays showed, in HKCs and HeLa cells, that both Klf4 and Sp3 bind the GC rich proximal region of the Notch 1 promoter (Fig. 7A-D). Interestingly, in HKCs, Sp1 was also found to bind to this region, but to a much lesser extent than to the proximal region of the p21WAF1/Cip1 promoter, a well established Sp1 target [28]. No Sp1 binding to the Notch1 promoter was found in HeLa cells (Fig. 7B). Similar Chip assays were also performed with antibodies against an unrelated GC-binding transcription factor, Maz [29]. While Maz1 was found to bind to the p21 promoter similarly to Sp1, Sp3 and Klf4, there was little or no binding of this protein to the proximal region of the Notch1 promoter (Fig. 7B, C).

### 4. Opposite control of Notch1 transcription by p53 versus Klf4/Sp3

**Figure 8:**
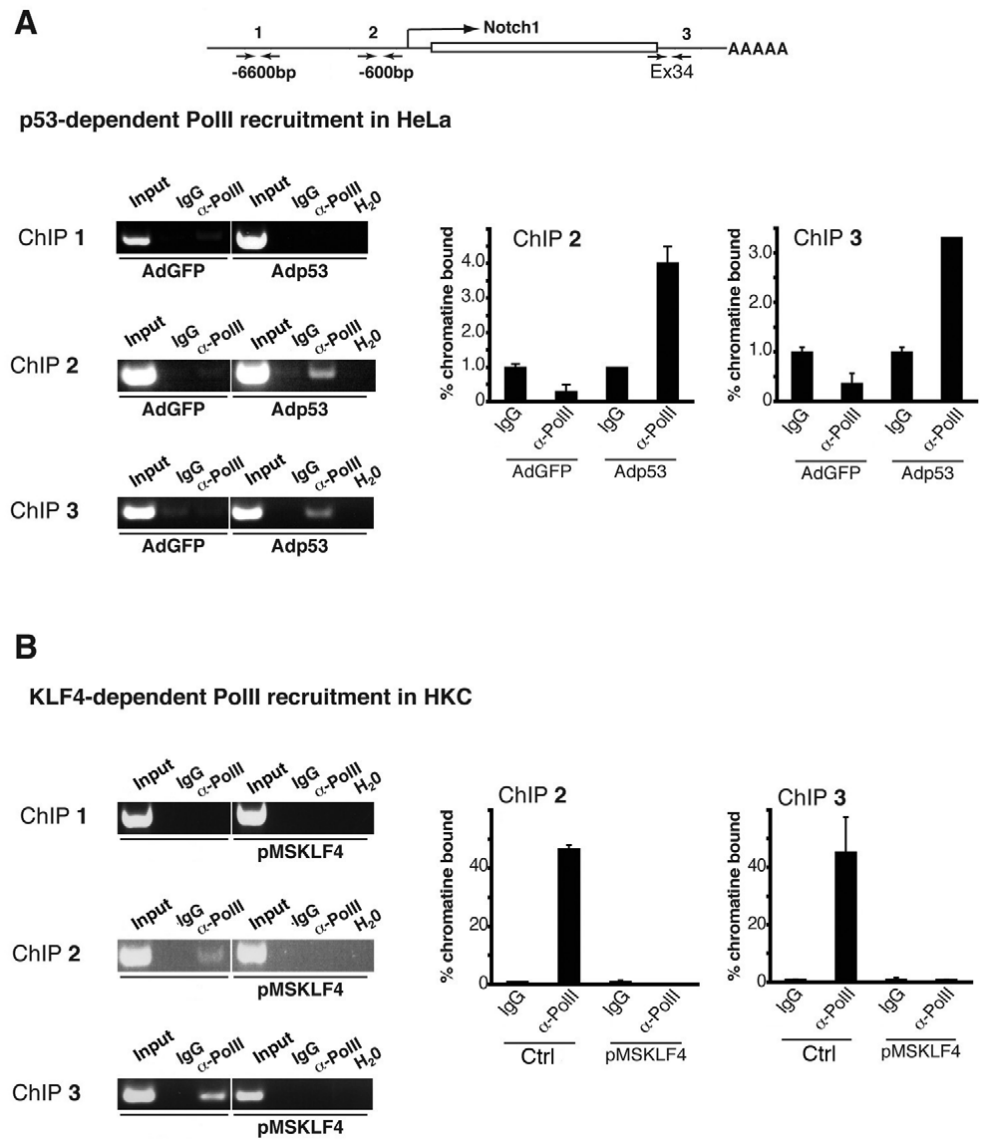
Opposite control of PolII recruitment to the Notch1 promoter by p53 and Klf4. (A) HeLa cells were infected with a recombinant adenovirus expressing wild-type p53 (Adp53) or GFP control (AdGFP) and processed for ChIP assays with antibodies against RNA polymerase II (α-PolII) and non-immune IgGs as control. PCR amplification of the indicated regions of the Notch1 gene was performed, in parallel with similar amplification of the input chromatin DNA. Right panels: for quantification of the results, the chromatin immunoprecipitated material was also analyzed by real time PCR amplification of the indicated regions of the Notch1 promoter, in parallel with input chromatin DNA, followed by a calculation of binding according to the same formula utilized in Fig. 7D. (B) Primary keratinocytes (HKC) were infected with a retroviral vector over-expressing Klf4 (pMSKlf4) or empty vector control (Ctrl) for 48 hours followed by ChIP assays for PolII binding as in the previous panel, including quantification of the results by real time PCR (right panels).

An important question is whether p53 and Klf4/Sp3 control Notch1 gene transcription through separate or convergent mechanisms. A key regulatory step is transcription factor-dependent recruitment of the transcription pre-initiation complex, containing RNA Polymerase II (PolII), to target promoters [30]. To assess whether this initial step of Notch1 transcription is under p53 and Klf4/Sp3 control, two complementary approaches were undertaken. In HeLa cells, where p53 levels are very low due to E6-dependent degradation, we evaluated the effects of increased p53 by adenoviral-mediated over-expression. In control HeLa cells, ChIP assays showed little or no binding of PolII to the promoter and 3′UTR of the Notch1 gene, while such binding was readily detectable in cells with increased p53 expression (Fig. 8A). In HKC, where levels of Klf4 are normally low, we evaluated the effect of increased Klf4 expression via retroviral infection. ChIP assays showed binding of PolII to the promoter and the 3′UTR of the Notch1 gene in control HKCs, while no such binding was detectable in cells with increased Klf4 expression (Fig. 8B).

**Figure 9:**
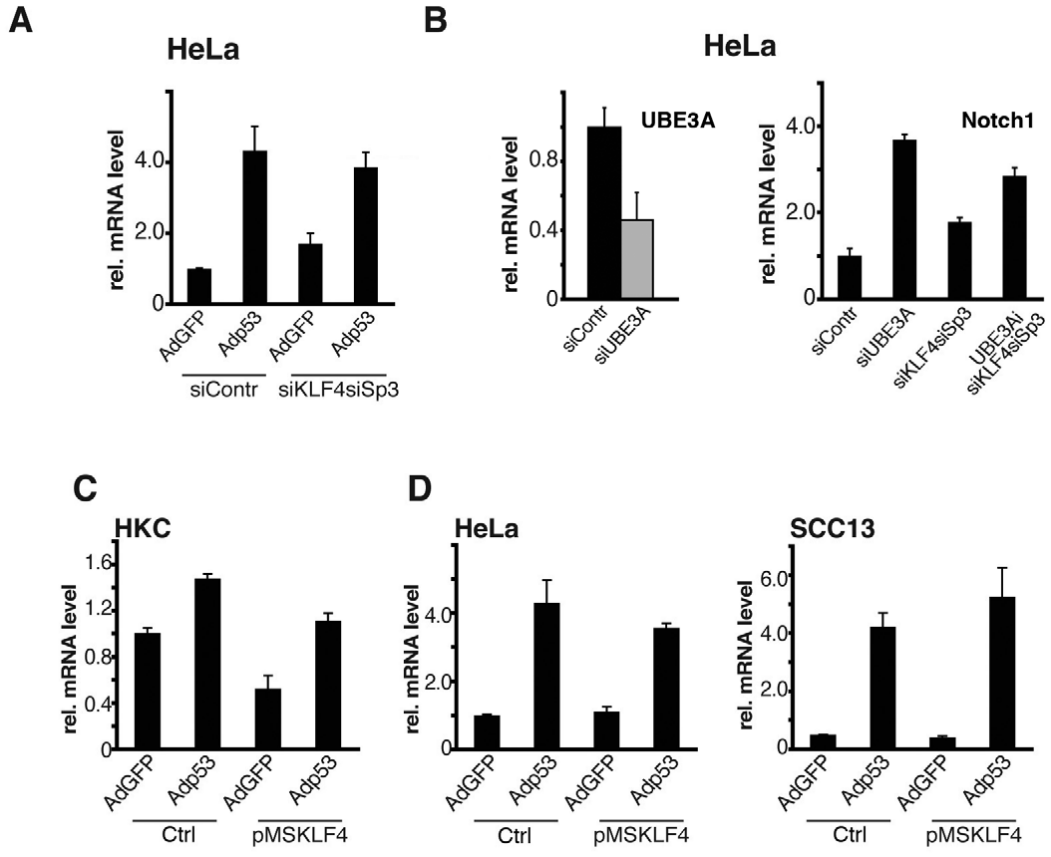
Separate control of Notch1 transcription by Klf4/Sp3 and p53. (A) HeLa cells were transfected with siRNAs for Sp3 and Klf4 in parallel with scrambled siRNA controls followed, 48 hours after transfection, by infection with a p53 expressing adenovirus (Adp53) or GFP control (AdGFP), for 24 hours. Levels of Notch1 mRNA expression were determined by real-time RT-PCR. The expected changes of p53, Sp3 and Klf4 expression were also confirmed by real time RT-PCR, with results similar to those shown in previous figures. (B) HeLa cells were transfected with siRNAs for UBE3A, Klf4 and Sp3 alone or in combinations as indicated, in parallel with scrambled siRNA controls. UBE3A and Notch1 expression was assessed by real-time RT-PCR. (C and D) Primary keratinocytes (HKC) (C) and HeLa and SCC13 cells (D were infected with a Klf4 expressing retrovirus or empty vector control for 48 hours, followed by infection with the Adp53 or AdGFP viruses for 24 hours. Notch1 mRNA levels were assessed by real-time RT-PCR.

Functionally, to assess whether increased p53 expression and Klf4/Sp3 down-modulation can synergize, two complementary approaches were undertaken. In the first, HeLa cells were infected with a p53 expressing adenovirus plus/minus knock-down of Klf4 and Sp3 expression. In the second, HeLa cells were transfected with siRNAs specific for the UBE3A protein, a negative regulator of p53 stability, as a method to increase endogenous p53 expression [14]. Increased p53 levels by either approaches caused the expected induction of Notch1 expression. The combined knockdown of Klf4 and Sp3 caused also an increase of Notch1 expression, but no additive or synergistic effects were observed by the concomitant increased of p53 and the knock-down of Klf4 and Sp3 (Fig. 9A and B).

To assess whether Klf4 can suppress the inductive effects of p53, HKCs, HeLa and SCC13 cells were infected with the Klf4 expressing retrovirus or empty vector control, followed by infection with the p53 expressing adenovirus or GFP control. Increased p53 levels caused induction of Notch1 expression in control HKCs as well as in HKCs where the Notch1 gene was down-modulated as a consequence of increased Klf4 expression (Fig. 9C). A more substantial induction of Notch1 expression was caused by Ad-p53 infection of cancer cells, like HeLa and SCC13 cells, which share a mutated or silent p53 pathway and low endogenous Notch1 levels [9], [11]. In these cells, which express high level of endogenous Klf4, further expression of Klf4 did not decrease Notch1 expression and did not interfere with its induction by p53 (Fig. 9D).

Thus, p53 and Klf4 converge on control of Notch1 gene transcription at the initial step of PolII recruitment, with p53 induction occurring through a separate mechanism from Klf4/Sp3 repression.

## Discussion

Tumor suppressor genes are classically defined as genes whose mutation or loss is required for tumor development [31]. The importance of transcriptional down-regulation of these genes is being increasingly recognized [32]. In specific cell types like keratinocytes, Notch signaling plays an important pro-differentiation and tumor suppressing function [3], [33], which is associated with specific down-modulation of the Notch1 gene in keratinocyte-derived cutaneous squamous cell carcinomas (SCCs) [9] and basal cell carcinomas (BCCs) [10], as well as in late stage cervical carcinomas, another important form of keratinocyte-derived tumors [11], [14]. Besides being controlled by p53 in keratinocytes and other cell types where Notch1 may play a growth/tumor suppressing function [34], little else is known on regulation of Notch1 gene expression. Very recently, negative control of Notch1 transcription by the Id3 repressor has been implicated as a requisite step for T cell maturation [35]. We have shown here that transcription of this gene is driven by a TATA-less “sharp peak” promoter and that the minimal functional region of this promoter, which extends from the -342 bp position to the initiation codon, is differentially active in normal versus cancer cells. This GC rich region lacks p53 binding sites, but binds Klf4 and Sp3. This finding is likely to be of biological significance, as Klf4 and, to a lesser extent, Sp3 are up-regulated in a number of cancer cells where Notch1 expression is down-modulated, and Klf4 over-expression in normal cells is sufficient to down-modulate Notch1 gene transcription. The combined knock-down of Klf4 and Sp3 was necessary for the reverse effect of increasing Notch1 transcription, consistent with the two factors exerting an overlapping repressor function through their binding to the Notch1 promoter.

Regulation of transcription occurs at multiple levels, with recruitment of the transcriptional machinery to the initiation site being a key step [30]. We have shown here that p53 and Klf4/Sp3 converge on recruitment of PolII to the Notch1 promoter, with one promoting and the other suppressing this event. The human Notch1 promoter region contains different putative p53-binding sites, which consist of single and double half-sites, each composed of two quarter-sites with a head-to-head orientation [36] (Fig. 1). Besides the quarter-site orientation, another factor affecting strength of p53 regulation is the distance between its cis-element binding elements and the transcription start site. DNA looping allows tetramer formation by p53 bound to distant and proximal promoter sites, thereby enhancing the impact on transcription. In the absence of proximal p53 binding sites, association with other proteins may mediate the juxtaposition of p53 to the transcription initiation complex [36]. A well established example of a p53 bridging protein is Sp1 [37], which is antagonized by Sp3, in a gene and promoter specific manner [27]. An interesting possibility was that of a p53/Sp1 versus Klf4/Sp3 antagonism applying to control of the Notch1 promoter proximal region that we have studied. However, even in normal cells, Sp1 was found to bind to this region to a much lesser extent than to the p21 proximal promoter, a well established Sp1 binding region [28]. In addition, knock down of Sp1 had no effect on basal levels of Notch1 gene transcription or its induction by p53. Conversely, the combined knock down of Klf4 and Sp3 resulted in increased Notch1 expression not only in normal keratinocytes but also in cancer cells like SCC13 and HeLa, where p53 is mutated and/or very lowly expressed. Thus, control of Notch1 gene transcription by Klf4 and Sp3 is likely to occur through a separate parallel mechanism from that by p53.

While originally identified for its general transcription housekeeping function, genetic studies have demonstrated that Sp1, and its related Sp3 family member, play a surprisingly specific and essential function during development [38], [39]. In keratinocytes, these factors have been implicated in control of differentiation marker genes like involucrin [40], loricrin [41] and SPRR [42] as well as p21 [28], which, besides its role in the cell cycle, plays a crucial role in the differentiation-associated transcription program [43]. In these cells, as in other contexts, Sp1 and Sp3 can play distinct regulatory functions, with Sp1 acting as an activator, and Sp3 as either an activator or repressor of transcription.

Like Notch1, Klf4 plays a highly context-dependent function in growth/differentiation control, being capable of both promoting and suppressing these processes even within the same cell type, depending on stages of development and in normal versus pathological conditions [20]. The dual function of this factor has been well demonstrated in the keratinocyte/skin system. Genetic deletion experiments have showed that Klf4 plays an essential positive role in the late steps of keratinocyte differentiation required for epidermal barrier formation [21]. However, Klf4 is also often over-expressed in keratinocyte-derived squamous cell carcinomas [44], and initial stages of carcinogenesis [45], with keratinocyte-specific transgenic expression promoting skin tumor formation [46]. Other tumor types have also been variously associated with increased or decreased Klf4 expression and function [20]. Recently, in RK3E cells, an E1a immortalized rat kidney cell line in which both Klf4 and Notch1 activation lead to transformation, Klf4 was shown to induce Notch1 expression [47]. The underlying mechanism involves also, as we have shown here, direct binding of Klf4 to the Notch1 promoter [47], pointing to the existence of additional cell type specific determinants of Klf4 function as either positive or negative regulator of Notch1 transcription.

A common feature of transcription networks is the presence of feedback regulatory loops used for amplification of an initial signal through positive reinforcement, or the dampening or termination of a signaling cascade, through negative regulation [48]. Thus, Notch1, besides being a downstream target of Klf4, may also be involved in control of this latter molecule. This appears to be the case in intestinal goblet cells in which Klf4 expression, essential for differentiation, is under negative Notch control [49], [50], [51]. In our system, activated Notch1 expression did not exert any significant effects on Klf4 expression (data not shown), but this may still occur under other experimental conditions and/or *in vivo*.

Biologically, inhibition of Notch1 expression by increased Klf4 levels is consistent with the essential role of the latter factor in late stages of keratinocyte differentiation [21], where expression of the Notch1 gene needs to be turned off [7], [8]. However, Notch1 expression can also become aberrantly suppressed by Klf4 during cancer development. As such, our findings indicate that the same regulatory mechanisms that trigger the final stages of the terminal differentiation process can also promote cancer development by suppressing the intermediate commitment stages (for which Notch1 is required).

## Materials and Methods

### Cells and viruses

Primary human keratinocytes and cancer cell lines were cultured as previously described [22]. The Ad-p53 virus was a gift of Dr. S. Lee (Massachusetts General Hospital, Charleston, MA), and the pMX-Klf4 retroviral vector [52] was obtained from Addgene. Conditions for adeno- and retro-virus production and infection, and for siRNA transfection were as previously described [9].

### Plasmids

The Notch1 promoter regions −2472/−1 and −392/−1 (numbered relative to the ATG) were amplified by PCR from HKs or HeLa (referred as −392 (D-215-202) in the text) genomic DNA with the primer pairs 5′-CTGCCTCCCGACCTGTAGGAG-3′ and 5′-GCCTCCCCACCGGCTGCCCTC-3′, and 5′-CTCGGGGAGGCGCAAAGGCGG-3′ and 5′-GCCTCCCCACCGGCTGCCCTC-3′, and subcloned into the pGL4 vector (Promega) using the KpnI/NheI sites. The −358/−1, −342/−1, −315/−1 and −300/−1 Notch1 promoter regions were amplified by PCR from the −392pGL4 clone using the reverse primer 5′-GCCTCCCCACCGGCTGCCCTC-3′ in combination with the following forward primers: 5′-GAGGAGGTGGCCGCGGAG-3′, 5′-agggggcggggggaccggcg-3′, 5′-ggccgggcggggcggggccggc-3′ and 5′-ggccggcgggggcggagcgcacc-3′ respectively. The inserts were subsequently subcloned in the pGL4 vector using the KpnI/NheI sites. The −530/−1 Notch1 promoter region was isolated using the previously cloned −976/−1 Notch1 promoter region cut with BamHI and NcoI and subcloned in the pGL4 vector through BglII/NcoI sites. The −530/−159 fragment was derived from the −530/−1 clone, by cutting with KpnI and SmaI, and inserted in the pGL4 vector using the KpnI/EcoRV sites. The −530/−262 fragment was derived from the −530/−1 clone, by cutting with KpnI and SpeI, and inserted in the pGL4 vector using the KpnI/NheI sites. The human cDNA clone for Klf4 was obtained from Origene (TC117491).

### RACE

Total RNA was treated with calf intestinal pyrophosphatase (CIP) to remove the 5′ phosphates of truncated mRNA, and with tobacco acid pyrophosphatase (TAP) to remove the 5′ cap structure from intact, full-length mRNA. GeneRacer RNA Oligo (Invitrogen) was ligated to the 5′ end of the mRNA using T4 ligase. Reverse transcription using random primers created RACE-ready first-strand cDNA with known priming sites at the 5′ end. The first-strand cDNA was amplified later on with the GeneRacer 5′ Primer (homologous to the GeneRacer RNA Oligo) and the reverse gene-specific primer located at the position +242 bp for the downstream transcription (5′-CTCTGCGGTCCACCACGTGGCATGT-3′) and at the position -1136 bp for the upstream transcription (5′-GTGCCTGGCATCGTGGTGGAGAA-3′). RACE PCR products were purified using S.N.A.P. columns and run on an agarose gel. The bands were cut, cloned into the pCR4-TOPO vector (Invitrogen) and subsequently sequenced.

### Promoter activity assays

Cells were plated onto 12-well dishes and transfected with 2 mg of luciferase reporter plasmid and 20 ng of phRLTK (Promega) as internal control, using Lipofectamine 2000 (Invitrogen) according to the manufacturer's instructions. Cells were lysed with Passive Lysis Buffer, and luciferase activity measured with Dual-Luciferase/Renilla Reporter Assay System (Promega) 48 hours after transfection. All conditions were tested in triplicate wells and relative values were normalized for Renilla luciferase activity. Lysate proteins were quantified with Bradford reagent as a second normalization. All experiments were repeated at least three times.

### Real time RT-PCR

Conditions for total cell RNA preparations and real time RT-PCR were as previously described [9], using an Icycler IQ Real-Time Detection System (Bio-Rad) according to the manufacturer's recommendations, with SYBR Green (Bio-Rad) for detection. Amplification of the same cDNAs with human 18S or 36b4 primers was used for internal normalization. Total RNA was extracted and purified from different cell types and conditions with TRI-reagent (Sigma) and DNase I treated following the manufacturer's instructions. 1 µg of total RNA was used to synthesize cDNA with random hexamer primers and SuperScript RNAse H- Reverse Transcriptase (Invitrogen). cDNA production was monitored with control β-actin PCR amplification. Real-time PCR was performed. Each sample was tested in triplicate with gene-specific primers (see supporting information, Table S1).

### Chromatin immunoprecipitation (ChIP)

HKCs and HeLa cells were cross-linked in 1% formaldehyde for 5 and 10 min respectively. Nuclei were extracted by incubation of cells for 5 min using cold L1 solution (50 mM Tris pH 8, 2 mM EDTA, 0.1% NP-40, 10% glycerol) followed by treatment with L2 solution (1% SDS, 5 mM EDTA, 50 mM Tris pH 8). Nuclei were sonicated so that the average length of chromosomal DNA became 200–1000 bp. 1 ml of chromatin solution was precleared with 80 µl of 50% protein G–Sepharose slurry pretreated with 1 mg/ml sonicated salmon sperm DNA. DNA/protein complexes were precipitated by overnight incubation with 8 µg anti-Sp1 (PEP-2, Santa Cruz Biotechnology), 4 µg anti-Sp3 (D-20, Santa Cruz Biotechnology), 4 µg anti-Klf4 (H-180, Santa Cruz Biotechnology), 4 µg anti-PolII (N-20; Labforce) and 4 µg anti-MAZ (H-50, Santa Cruz Biotechnology) antibody or 4 µg normal rabbit anti-IgG antibodies as control (Santa Cruz Biotechnology). DNA/protein complexes were incubated with 30 µl of 50% protein G–Sepharose pretreated with salmon sperm DNA, followed by reversal of cross-links and deproteination. After phenol–chloroform extraction and ethanol precipitation, the recovered DNA was analyzed by PCR amplification. Preliminary experiments with naked DNA were carried out to optimize PCR conditions for each target sequence. The promoter for the Notch1 human gene contains several Sp/KLF-binding sites. All PCRs for the GC-rich Notch1 promoter sequence were performed using GC-rich PCR kit system (Roche).

### Immunoblots and antibodies

Conditions for immunoblotting were as previously described [9]. The following antibodies were used: goat polyclonal anti-Notch1 (C-20; Santa Cruz Biotechnology), rabbit polyclonal anti-Sp1, anti-Sp3, anti-Klf4 (Santa Cruz Biotechnology), anti-PolII and mouse monoclonal anti-γ-tubulin (Sigma), secondary HRP-conjugated antibodies (Amersham Bioscience). Signal was detected using SuperSignal West Pico Chemiluminescent Substrate (Pierce).

## Supporting Information

Table S1 List of primers for RT-PCR and real-time RT-PCR, and siRNAs (Stealth RNAi).
(0.07 MB PDF)
