# Supplementary material for: Differential Control of Notch1 Gene Transcription by Klf4 and Sp3 Transcription Factors in Normal versus Cancer-Derived Keratinocytes
Source: PLoS One. 2010 Apr 28;5(4):e10369. doi: 10.1371/journal.pone.0010369 (PMC2860992; doi:10.1371/journal.pone.0010369)
Supplement: Table S1 — List of primers for RT-PCR and real-time RT-PCR, and siRNAs (Stealth RNAi). (0.07 MB PDF) [file pone.0010369.s001.pdf]

Notch1 promoter RT-PCR primers:

Name	Primers sequence (5'-3')
**-740/-262**	Frontal: CACACACGGCTAGGCCAC<br>Reverse: GTGAGGCTCAGAGTCGAGGT
**-262/-174**	Frontal: ACTAGTGCCTCGGCCGCG<br>Reverse: CCCGGCTCGTTCCTTCGC
**-262/-68**	Frontal: ACTAGTGCCTCGGCCGCG<br>Reverse: ACACGCGCGGCGTACGGT
**+114/+1127**	Frontal: TCCGCGCCCTTTGGAAACTTT<br>Reverse: AGAATCAGAGCGGCCCATTGTG
**+1128/+1737**	Frontal: ACAATGGGCCGCTCTGATTCTG<br>Reverse: TTCACACTTCCCGCCATTCAGG

Real-time RT-PCR primers:

Name	Primers sequence (5'-3')
**Notch1 5'UTR/Ex3**	Frontal: AGAGGGCAGCCGGTGGGGAG<br>Reverse: TGCGGTCCACCACGTGGC
**Notch1 Ex33/3'UTR**	Frontal: CTGAAGAACGGGGCTAACAA<br>Reverse: CAGGTTGTACTCGTCCAGCA
**Notch1 Ex34**	Frontal: GAACCAATACAACCCTCTGC<br>Reverse: AGCTCATCATCTGGGACAGG
**Notch1 intron1**	Frontal: TTCCTGTTGCTTCTCCGGG<br>Reverse: AACCCCTCCCCCAAACTGA
**18S**	Frontal: GCAATTATTCCCCATGAACG<br>Reverse: GGCCTCACTAAACCATCCAA
**36b4**	Frontal: GCAATGTTGCCAGTGTCTGT<br>Reverse: GCCTTGACCTTTTCAGCAAG
**Sp1**	Frontal: GCTGGAGAGTCAAAGGCAT<br>Reverse: TAGTGAAAGCCCCCTACC
**Sp3**	Frontal: GTTGAGGCATTTGGGTGGTA<br>Reverse: GGGAGACATGGTTTTTGGAA
**KLF4**	Frontal: CCAATTACCCATCCTTCCTG<br>Reverse: CGATCGTCTTCCCCTCTTTG
**KLF5**	Frontal: CCCTTGCACATACACAATGC<br>Reverse: AGTTAACTGGCAGGGTGGTG
**KLF10a**	Frontal: CAACTTCGGTGCCTCTCT<br>Reverse: GCTCATTGACATAAGTGCTTCT

ChIP primers:

Name	Primers sequence (5'-3')
**Site '1'(-6669/-6113)**	Frontal: GGGAAGAGAGAGTGCAGCTGC<br>Reverse: TGCCTGGTGCCCCAAGTGG
**Site '2' (-740/-262)**	Frontal: CACACACGGCTAGGCCAC<br>Reverse: GTGAGGCTCAGAGTCGAGGT
**Site '3'(Ex34)**	Frontal: GAACCAATACAACCCTCTGC<br>Reverse: AGCTCATCATCTGGGACAGG
**ChIP p21**	Frontal: CCGGCTCCACAAGGAACT<br>Reverse: ACCAACGCAGGCGAGGGACT

siRNAs:

Name	Oligo ID	Provider
Sp1i	HSS110104	Invitrogen
Sp3i	HSS110111	Invitrogen
KLF4i	HSS113794	Invitrogen
UBE3Ai	HSS111136	Invitrogen

Table S1. List of primers for RT-PCR and real-time RT-PCR, and siRNAs (Stealth RNAi)
